# Supplementary figures and images for: Glycosylated Delphinidins Decrease Chemoresistance to Temozolomide by Regulating NF-κB/MGMT Signaling in Glioblastoma
Source: Cells. 2025 Jan 24;14(3):179. doi: 10.3390/cells14030179 (PMC11816850; doi:10.3390/cells14030179)

(a)

DMSO

DEL3

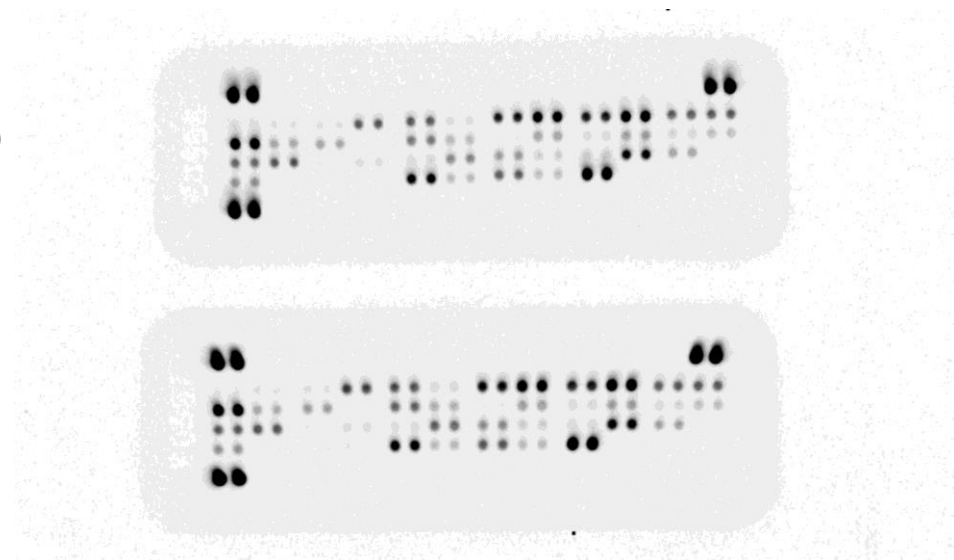

(b)

DMSO

DEL3,5

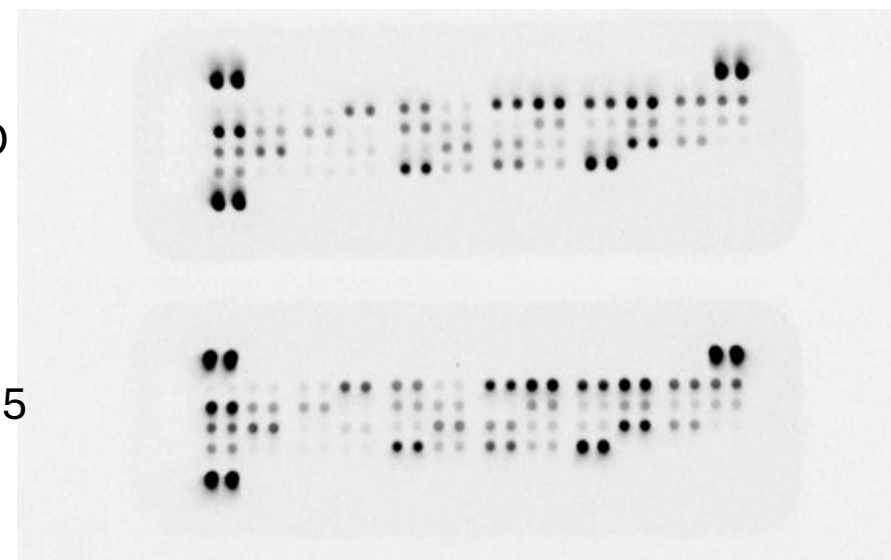

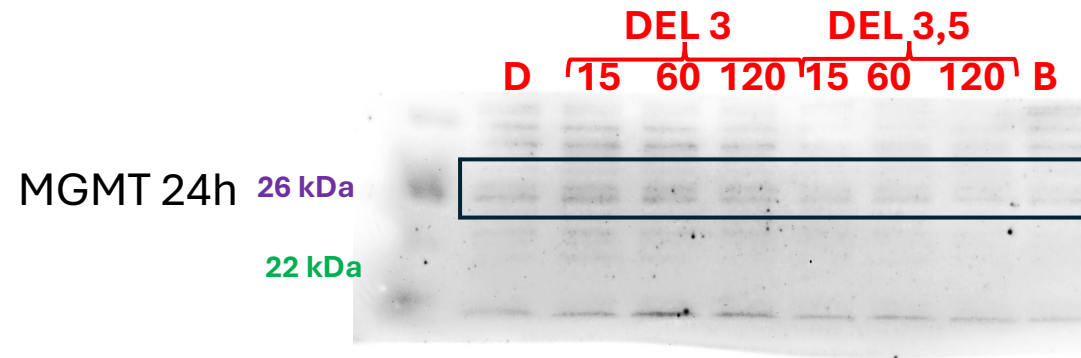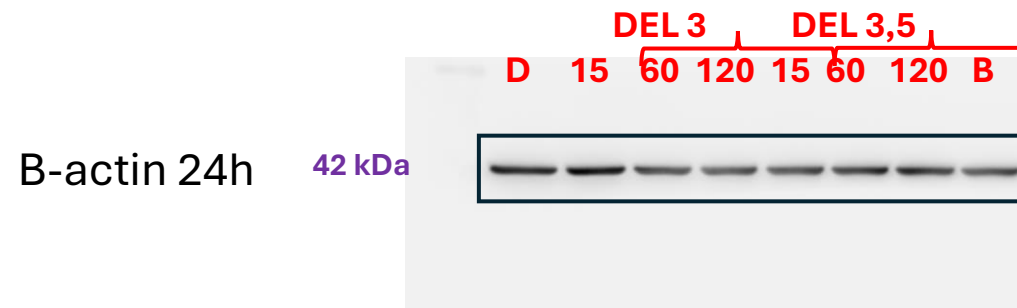

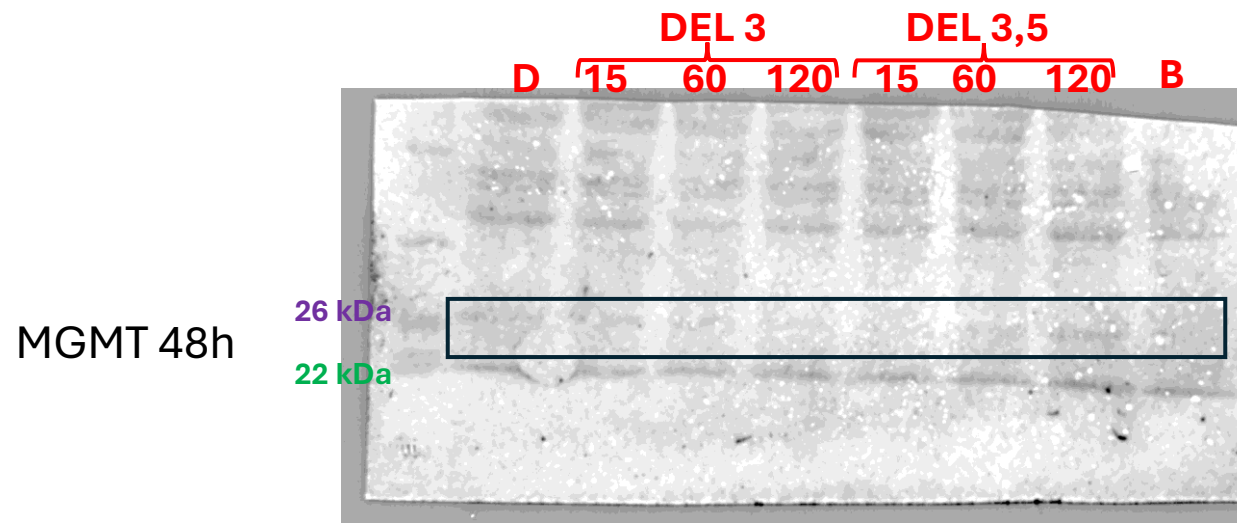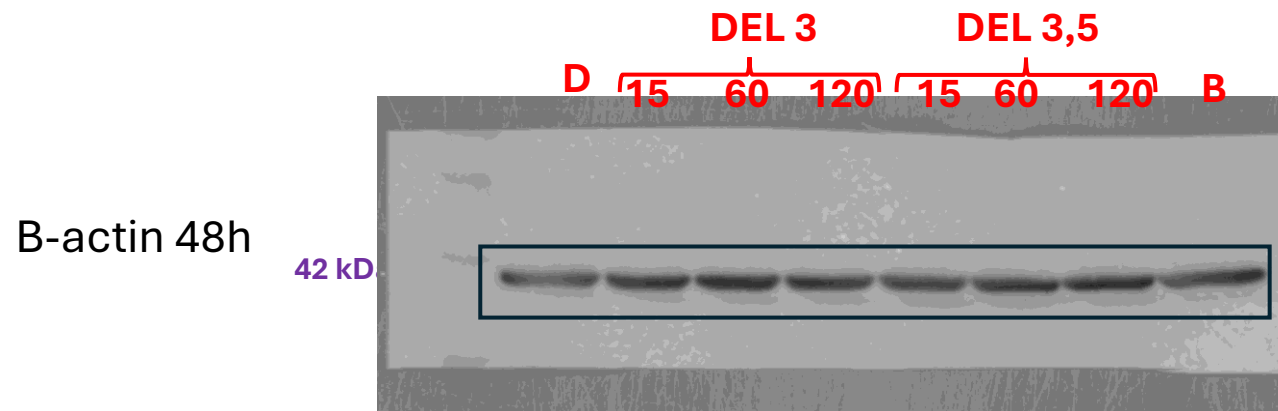

Supplement: Supplementary file 1 [file cells-14-00179-s001.zip › cells-3399892-supplementary.pdf]
